# Curcumin Improves Cardiopulmonary Resuscitation Outcomes by Modulating Mitochondrial Metabolism and Apoptosis in a Rat Model of Cardiac Arrest

**DOI:** 10.3389/fcvm.2022.908755

**Published:** 2022-05-19

**Authors:** Jie Zhang, Siqi Liu, Longyuan Jiang, Jingying Hou, Zhengfei Yang

**Affiliations:** Sun Yat-sen Memorial Hospital, Sun Yat-sen University, Guangzhou, China

**Keywords:** cardiac arrest, cardiopulmonary resuscitation, mitochondria metabolism, myocardial dysfunction, apoptosis, curcumin

## Abstract

**Background:**

Curcumin, a diarylheptanoid chemical compound extracted from curcuma longa, exerts a variety of biological and pharmacological effects in numerous pathological conditions, including ischemia/reperfusion (I/R) injury. In this study, we investigated its role in post-resuscitation myocardial dysfunction in a rat model of cardiac arrest (CA) and cardiopulmonary resuscitation (CPR) by targeting on mitochondrial metabolism and apoptosis.

**Methods:**

Animals were randomized into three groups: sham, control and curcumin, with fifteen rats in each group. Ventricular fibrillation (VF) was induced in the rats of the control and curcumin groups. The rats in the two groups were untreated for 8 min, followed by CPR for 8 min. Placebo (saline) or curcumin was administered by intraperitoneal injection, respectively, 5 min after successful resuscitation. Myocardial function was measured at baseline and post-resuscitation for 6 h consecutively. Ten rats in each group were closely observed for an additional 66 h to analyze the survival status, and the remaining five were sacrificed for the measurement of mitochondrial parameters and cell apoptosis.

**Results:**

Compared with the control group, myocardial function was significantly enhanced in the curcumin group, contributing to a better survival status. Curcumin treatment mitigated the depletion of superoxide dismutase (SOD) and the production of malondialdehyde (MDA). The structural damage of mitochondria was also alleviated, with improved conditions of mPTP and ΔΨm. Curcumin boosted the production of ATP and attenuated myocardial apoptosis. Cytochrome C, caspase-3 and its cleavage were suppressed by curcumin. Proteins closely related to the functional performance of mitochondria, including uncoupling protein 2 (UCP2) and uncoupling protein 3 (UCP3) were downregulated, while mitochondrial transcription factor A (mtTFA) was upregulated.

**Conclusion:**

Curcumin improves the outcomes of CPR *via* alleviating myocardial dysfunction induced by I/R injury. It exhibits anti-oxidation properties. Moreover, it is capable of ameliorating mitochondrial structure and energy metabolism, as well as inhibiting the mitochondrial apoptosis pathway. UCP2, UCP3, and mtTFA might also be involved in curcumin mediated protective effects on mitochondria.

## Introduction

Cardiac arrest (CA) is a severe public health problem with high morbidity and mortality. The incidence of out-of-hospital CA in adults is 95.9/100,000/year on average worldwide (1). Cardiopulmonary resuscitation (CPR) is the most widely applied treatment for CA in clinical practice (2, 3). However, even with prompt intervention and successful resuscitation, the overall survival rate of CA is less than 5%, mainly due to post-resuscitation myocardial dysfunction, neurological dysfunction and inflammation resulting from ischemia/reperfusion (I/R) injury (4, 5). Currently, accumulating evidence has demonstrated that mitochondria are the prime targets of I/R injury, ascribing to their significant role in reactive oxygen species (ROS) generation (6, 7).

Curcumin is a yellow diarylheptanoid chemical compound isolated from the therapeutic and dietary spice turmeric (*Curcuma longa*). As the active component in turmeric, curcumin could exert multifarious biological and pharmacological effects, such as anti-inflammation, anti-oxidation, anti-thrombosis, anti-tumorigenesis, chemopreventive and chemotherapeutic properties, etc. (8, 9). In addition, it is non-toxic and affordable even when adopted in high doses. Due to these superiorities, curcumin has become a unique candidate for drug development in various diseases (9).

The underlying mechanisms of curcumin in the prevention and treatment of I/R injury have been investigated in a variety of pathological conditions. Previous studies have validated its effects on ROS production, endogenous antioxidant enzymes, and mitochondrial function (10–12). However, the influence of curcumin on myocardial injury after CA and CPR remains not entirely clear. Successful restoration of spontaneous circulation (ROSC) post CPR signifies the reestablishment of aerobic metabolism. Mitochondria are the centers of energy metabolism, and they are specifically important in regulating cell death and apoptosis provoked by I/R injury (6, 7, 13). In this condition, we aimed to investigate the protective properties of curcumin against post-resuscitation myocardial damage in a rat model of CA induced by ventricular fibrillation (VF). The relevant underlying mechanisms were further explored by focusing on its role in mitochondrial metabolism and apoptosis. This study could provide an experimental basis for the application of curcumin as a potential strategy in treating myocardial dysfunction after successful resuscitation in CA patients.

## Materials and Methods

Experiments were performed with the approval of the Institutional Animal Care and Use Committee of the Tang Wanchun Laboratories of Emergency Critical Care Medicine, Sun Yat-sen Memorial Hospital of Sun Yat-sen University. The rat model of CPR established by the following preparations was utilized in this study (14–16). Baseline animal characteristics were obtained 15 min before operating VF. Forty-five rats were randomly assigned into three groups, with fifteen rats in each group. For the curcumin and control groups, curcumin or placebo (saline) was treated, respectively, at 5 min after successful resuscitation. Curcumin dissolved in DMSO was administered by intraperitoneal injection at a dose of 100 mg/kg (17, 18). The sham group did not undergo CA and any treatments. Ten rats in each group were randomly selected for the survival analysis, and the survival status of the three groups was recorded afterward. The remaining five were euthanized to obtain tissues for the detection of relevant parameters.

### Animal Preparation

Male Sprague-Dawley rats (6–8 months, 450–550 g) from the Experimental Animal Center of Traditional Chinese Medicine University of Guangzhou were used. Anesthesia and trachea intubation was performed as our previous published work (14–16). All the animals received a subcutaneous injection of buprenorphine (1 mg/kg) followed by monitoring. The end-tidal CO_2_ (ETCO_2_) and conventional lead II electrocardiogram were continuously monitored to ensure the spontaneous breath of the rats (14–16).

For measurements of aortic pressure and blood gas, a polyethylene catheter (PE-50; Becton Dickinson, Franklin Lakes, NJ, United States) was inserted into the descending aorta from the surgically exposed left femoral artery. Methods of the right atrial pressure detection and VF induction were all prepared as previously (14–16). A thermocouple microprobe (9030-12-D-34; Columbus Instruments, Columbus, OH, United States) was advanced into the left femoral vein to monitor the blood temperature. The temperature was maintained at 37 ± 0.5°C by a cooling blanket or infrared surface heating lamps (14–16).

### VF Induction and CPR

Mechanical ventilation was established, and VF was induced as previously described (14–16). After 8 min of untreated VF, precordial compression and mechanical ventilation were initiated concurrently. Precordial compression was conducted as previously (14–16). The depth of compression was initially adjusted to maintain a coronary perfusion pressure (CPP) of 22 ± 2 mm Hg (19). The procedure of epinephrine administration and defibrillation was also performed thereafter as previously reported (14, 15, 20). ROSC was achieved when the supraventricular rhythm was resumed, with a mean aortic pressure of greater than 50 mm Hg for a minimum of 5 min. Mechanical ventilation was continued after ROSC as recorded (14, 15, 20). Six hours post resuscitation, ten rats in each group were closely observed for an additional 66 h to analyze the survival status (14, 15, 20). The rest five rats were sacrificed to obtain the left ventricular (LV) wall tissues for the determination of mitochondria ultrastructure and relevant molecular parameters.

### Physiological Indexes Measurement

For the analysis of aortic and right atrial pressures, electrocardiogram data and ETCO_2_ values, a PC-based data-acquisition system supported by WINDAQ software was used (DATAQ, Akron, OH, United States). CPP was calculated as the difference between time-coincident decompression diastolic aortic and right atrial pressure displayed in real time (14, 15, 20). After ROSC, myocardial function-cardiac output (CO), ejection fraction (EF) and myocardial performance index (MPI) were non-invasively measured for 6 h as provided in our previous work (14–16). All the measurements of these indexes were reviewed and confirmed by two independent investigators separately. The measurement of aortic blood pH, partial pressure of carbon dioxid (P_*CO2*_), partial pressure of oxygen (P_*O2*_), hemoglobin and lactate concentrations were performed at different time points with a Stat Profile pHOx Plus L blood gas analyzer (Model RADIOMETER ABL80FLEX, Radiometer Medical ApS, Bronshoj, Denmark) (14, 15, 20).

### Examination of Mitochondria Ultrastructure

Myocardial tissue samples were fixed in 2.5% glutaraldehyde with 0.1 mol/L cacodylate buffer (pH 7.4) and post-fixed in 1% osmium tetroxide. Samples were subsequently dehydrated and embedded in epoxy resin. Ultrathin sections (60–80 nm) were stained with lead citrate and uranyl acetate. A FEI Tecnai G2 transmission electron microscope equipped with a Gatan 832 CCD camera (Gatan, Pleasanton, United States) was used to view these sections (21, 22).

### Detection of Mitochondrial Membrane Potential (ΔΨm) and Permeability Transition Pore

A tissue mitochondria isolation kit (C3606, Beyotime Institute of Biotechnology, Shanghai, China) was used to isolate the mitochondria from the free LV walls. The ΔΨm was measured by the MitoProbe JC-1 detection kit (#M34152; Thermo Fisher Scientific) according to the manufacturer’s instructions. Rat myocardial tissue slides were stained with 50 mM CCCP and 2 μM JC-1 at 37°C for 15 min, followed by the washing with PBS. The potential was measured using fluorescence microscopy. The opening of mitochondrial PTP (mPTP) was tested by the BestBio mPTP detection kit (#BB-48122; BestBio Science) at the absorbance of 490 nm according to the manufacturer’s instructions (21, 22).

### Measurements of Adenosine Triphosphate, Malondialdehyde and Superoxide Dismutase

ATP content in rat myocardial tissues was inspected using the ATP assay kit (#S0026; Beyotime, Beijing). Rat myocardial tissue homogenates in lysis buffer were centrifuged. The supernatants were mixed with ATP detection solution and kept at room temperature for 5 min. The levels of ATP were determined by measuring the relative light unit (RLU) with a luminometer, using the standard curve obtained from 0.01, 0.03, 0.1, 0.3, 1, 3 and 10 μM ATP samples as a reference. MDA levels were analyzed by the lipid peroxidation MDA assay kit (Colorimetric/Fluorometric) (#ab118970; Abcam) according to the manufacturer’s instructions. SOD activity was measured using the SOD assay kit (#19160; Sigma-Aldrich), as specified by the manufacturer (21, 22).

### Myocardial Apoptosis Evaluation

Myocardial apoptosis was evaluated by the TUNEL staining with the one-step TUNEL cell apoptosis detection kit (green fluorescence) (#C1088; Beyotime). Myocardial tissue slides were fixed with 4% paraformaldehyde for 30 min and washed twice with PBS for 10 min. Slides were incubated with 0.5% Triton X-100 in PBS for 5 min at room temperature, and stained with 50 ml TUNEL staining solution in darkness for 60 min at 37°C. Myocardial apoptosis was observed and calculated under the fluorescence microscopy as previously (22).

### Reagents

The following antibodies were used for western blotting analysis: rabbit monoclonal anti-uncoupling protein 2 (UCP2) (#89326, Cell Signaling Technology, United States; 1:1,000 dilution), rabbit polyclonal anti-uncoupling protein 3 (UCP3) (#BA1722, Boster Biological Technology, United States; 1:500 dilution), rabbit polyclonal anti-cytochrome C (Cyt C) (#ab90529, Abcam, United Kingdom; 1:1,000 dilution), rabbit monoclonal anti-mitochondrial transcription factor A (mtTFA) (#ab252432, Abcam, United Kingdom; 1:1,000 dilution), rabbit monoclonal anti-Cyt C oxidase subunit IV (COX IV) (#ab202554, Abcam, United Kingdom; 1:2,000 dilution), rabbit monoclonal anti-caspase 3 (#ab184787, Abcam, United Kingdom; 1:5,000 dilution), rabbit polyclonal anti-cleaved-caspase 3 (#9661, Cell Signaling Technology, United States; 1:1,000 dilution). Secondary antibodies and other reagents not specified were obtained from Sigma-Aldrich (Darmstadt, Germany).

### Western Blotting Analysis

Total proteins were extracted from the rat myocardium using the tissue or cell total protein extraction kit (#C510003; Sangon, Shanghai, China) according to the manufacturer’s instructions. Protein concentration measurements was performed using the BCA method. About 20 g of protein from each group was separated and resolved by 12% sodiun dodecyl sulfate polyacrylamide gel (SDS-PAGE). Quantified proteins were transferred to a polyvinylidene fluoride (PVDF) membrane before incubating with a 5% lipid-free milk solution for 2 h at room temperature. Proteins were then incubated with the primary antibodies, which were diluted as appropriately with TBST. The membranes were subjected to three washings with TBST and incubated with the secondary antibodies (Darmstadt, Sigma-Aldrich) for 60 min at room temperature. After extensive washing, the bands were detected by enhanced chemiluminescence intensities and quantified by using an image software (Image J, V.1.8.0, National Institutes of Health, United States) as previously (23, 24).

### Statistical Analysis

SPSS 16.0 (SPSS Inc., Chicago, IL, United States) was used for the statistical analysis. Quantitative data were described as mean ± standard deviation (SD) and analyzed by one way-ANOVA. Bonferroni test was applied for the comparison of the differences between the groups. Kaplan–Meier analysis was used for evaluation of the survival. A two-tailed *p* value less of 0.05 was considered to be statistically significant.

## Results

### Curcumin Improved Post-resuscitation Myocardial Function and Ameliorated Survival Status

**FIGURE 1:**
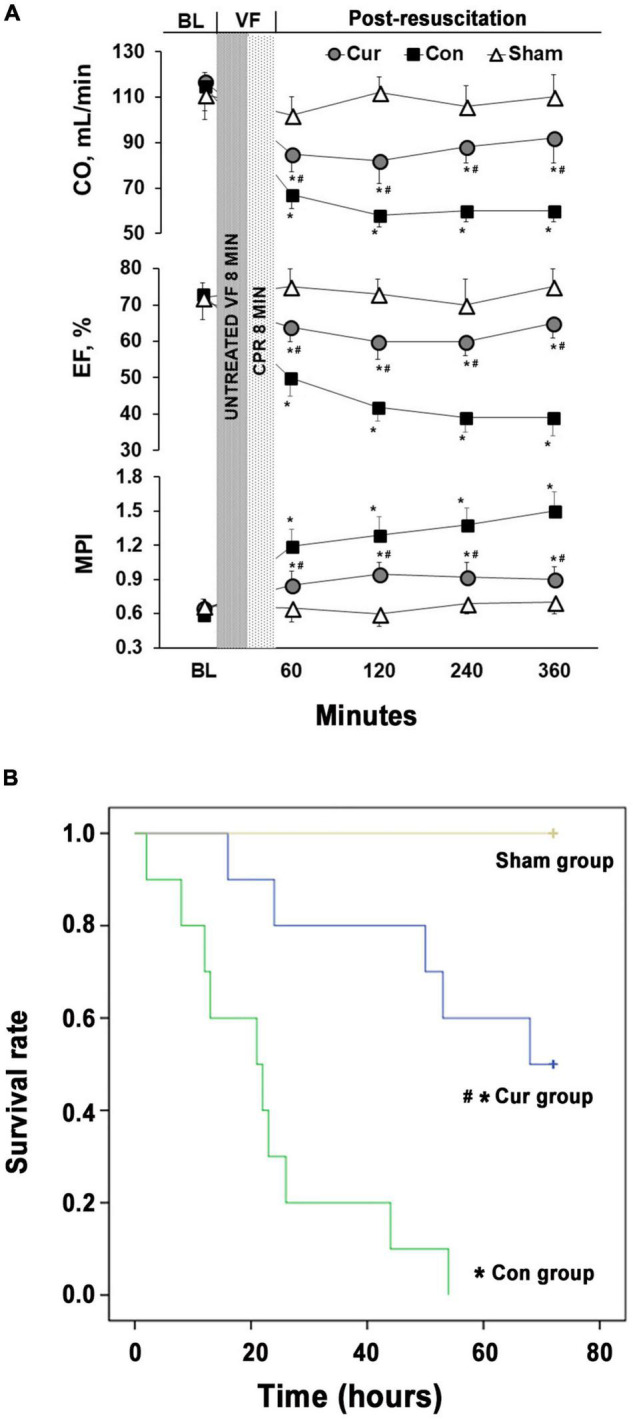
Curcumin ameliorated post-resuscitation myocardial dysfunction and prolonged survival duration. **(A)** The CO, EF and MPI were monitored for 6 h after CPR to evaluate post-resuscitation myocardial dysfunction. **(B)** Survival rates of the control, curcumin and sham groups were measured for 72 h after CPR. BL: baseline; VF: ventricular fibrillation; CO: cardiac output; EF: ejection fraction; MPI: myocardial performance index; CPR: Cardiopulmonary resuscitation; Cur: curcumin; Con: control. *p < 0.05 vs. the Sham; ^#^*p* < 0.05 vs. the Con.

There were no differences in baseline physiological parameters among the three groups. All the animals were successfully resuscitated in the curcumin and control groups. Meanwhile, there were no differences in the selective indexes evaluating the outcomes of CPR between the two groups (Table 1). After successful resuscitation, EF, CO and MPI were used to estimate myocardial function. Compared with the sham group, myocardial function was severely impaired in the control group, while curcumin treatment could partially reverse the post-resuscitation myocardial damage (Figure 1A). The survival rate of the curcumin group was also higher than the control group (*P* < 0.05). Additionally, several rats in the curcumin group survived through the entire 72-h observation period, however, none were survived in the control group (Figure 1B).

**TABLE 1:** Baseline information and selective outcomes of CPR.

	*Cur*	Con	Sham
Weight, g	508 ± 14	496 ± 9	502 ± 12
Heart rate, beats/min	354 ± 12	366 ± 17	359 ± 15
Mean artery pressure, mm Hg	137 ± 16	139 ± 20	145 ± 18
PH	7.52 ± 0.12	7.49 ± 0.15	7.51 ± 0.11
PaO_2_, mm Hg	85 ± 10	88 ± 8	84 ± 6
Lactate, mmol/L	0.6 ± 0.3	0.6 ± 0.4	0.7 ± 0.3
CPP in PC1, mm Hg	23.9 ± 1.4	23.4 ± 1.2	/
CPP in PC8, mm Hg	25.3 ± 1.6	26.1 ± 1.5	/
Duration of CPR, second	489 ± 10	485 ± 17	/
DFs, n	2.1 ± 1.1	1.7 ± 0.3	/
VAs, n	18 ± 8	22 ± 10	/

*Values are presented as mean ± SD.DMSO: dimethylsulfoxide; Cur: curcumin; Con: control; CPP: coronary perfusion pressure; PC1:1 min after precordial compression; PC8: 8 min after precordial compression; CPR: cardiopulmonary resuscitation; DFs: defibrillations; VAs: numbers of episodes of ventricular arrhythmias within the first 5 min post-resuscitation.*

### Curcumin Exhibited Anti-oxidative Activities in Post-resuscitation Cardiomyocytes

**FIGURE 2:**
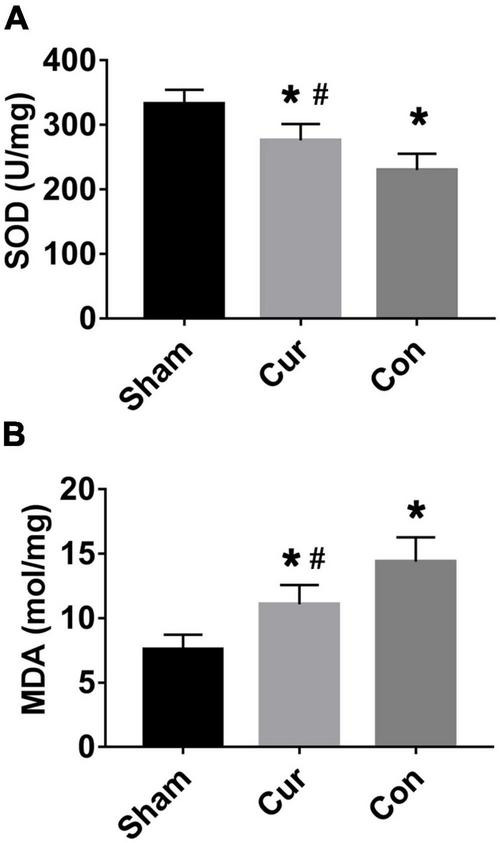
Curcumin protected post-resuscitation cardiomyocytes against oxidation stress. Values of SOD **(A)** and MDA **(B)** were detected in different groups. Cur: curcumin; Con: control; MDA: malondialdehyde; SOD: Superoxide dismutase; *p < 0.05 vs. the Sham; ^#^*p* < 0.05 vs. the Con.

Compared with the sham group, the SOD level was decreased in the curcumin and control groups, whereas the content of MDA was increased in the two groups (Figures 2A,B). The variation trend of SOD and MDA in the curcumin group was postponed in contrast with the control group (*P* < 0.05), revealing that curcumin treatment effectively retarded SOD depletion and obstructed MDA production in post-resuscitation cardiomyocytes (Figures 2A,B).

### Curcumin Improved Mitochondrial Ultrastructure and Function of Post-resuscitation Cardiomyocytes

**FIGURE 3:**
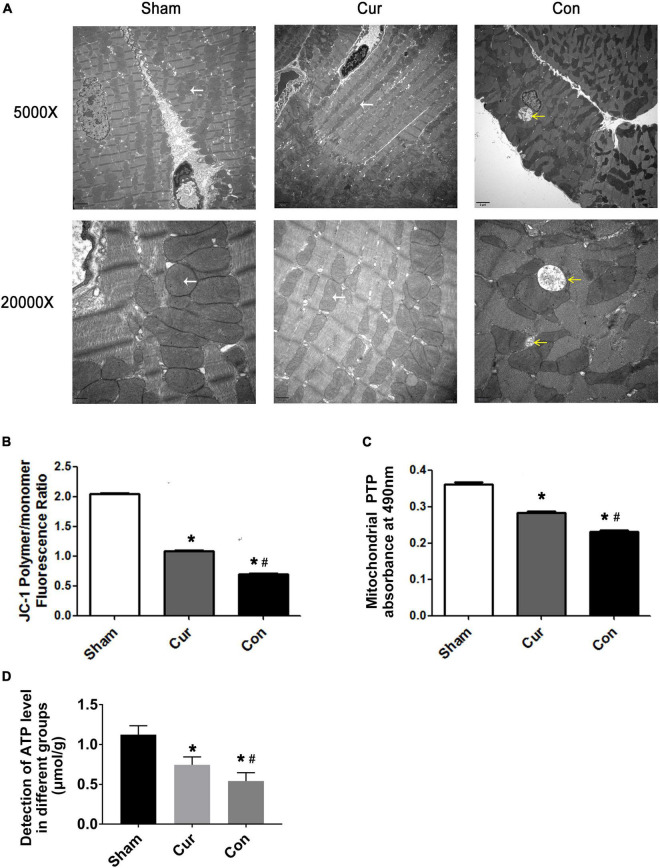
Curcumin improved mitochondrial structure integrality and energy metabolism. **(A)** Mitochondrial ultrastructure observed by transmission electronic microscope. The yellow arrows represented severely damaged mitochondria with swollen morphology, rarefied matrix and deformed cristae. The white arrows represented normal mitochondria. **(B)** Detection of the ΔΨm. **(C)** Detection of the opening of mPTP. **(D)** Detection of ATP level in different groups. Cur: curcumin; Con: control; ΔΨm: mitochondrial transmembrane potential; mPTP: mitochondrial permeability transition pores; ATP: adenosine triphosphate. *p < 0.05 vs. the Sham; ^#^*p* < 0.05 vs. the Con.

Mitochondrial ultrastructure and function after successful resuscitation was simultaneously assessed in this work. Sporadic changes were presented in the mitochondrial ultrastructure of the control group compared with the sham group, such as abnormal swelling of mitochondria, dissolution of cristae and reduced numbers of mitochondria (Figure 3A). However, curcumin administration obviously relieved deterioration of mitochondrial morphology and reduced the number of the injured mitochondria after resuscitation (Figure 3A).

The collapse of ΔΨ_*m*_ could be observed in both the curcumin and control groups. Curcumin administration extremely attenuated the reduction of ΔΨ_*m*_ induced by reperfusion (Figure 3B). Compared with the sham group, the opening of mPTP was widely induced in the control group, however, the mPTP opening was repressed in the circumstance of curcumin intervention (Figure 3C).

Moreover, ATP level was decreased in the control group, while its production was rehabilitated somewhat after curcumin treatment, indicating that curcumin might play a protective role in mitochondrial energy metabolism (Figure 3D).

### Curcumin Regulated the Expression of Mitochondrial Metabolism Related Proteins in Post-resuscitation Cardiomyocytes

**FIGURE 4:**
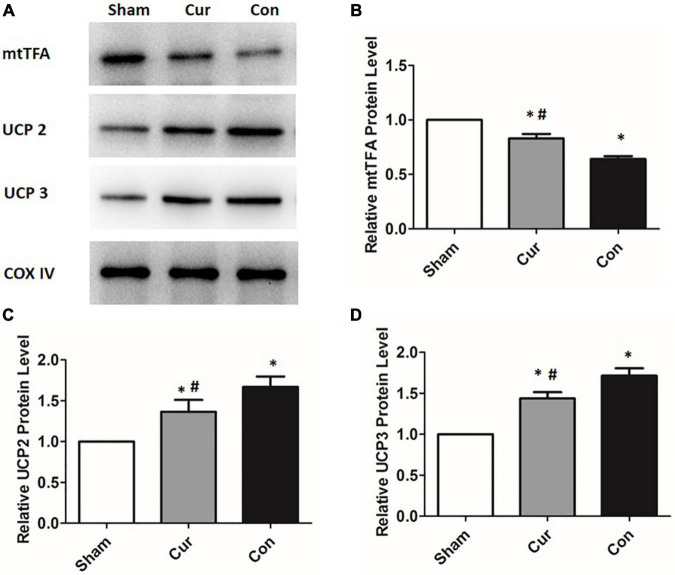
Curcumin treatment altered the expressions of mitochondrial proteins. Mitochondrial proteins including UCP2, UCP3 and mtTFA were determined by the western blotting assay. The COX IV was used as the internal control. **(A)** The blots of UCP2, UCP3 and mtTFA. **(B)** The relative expression of the protein level of UCP2. **(C)** The relative expression of the protein level of UCP3. **(D)** The relative expression of the protein level of mtTFA Cur: curcumin; Con: control; UCP2: mitochondrial uncoupling protein 2; UCP3: mitochondrial uncoupling protein 3; mtTFA: mitochondrial transcription factor A; *p < 0.05 vs. the Sham; ^#^*p* < 0.05 vs. the Con.

To explore the molecular mechanism of the effects mediated by curcumin, the expression of several key proteins associated with mitochondrial metabolism were further analyzed by western blotting. The expression of UCP2 and UCP3 in the control group was remarkably elevated after resuscitation (Figures 4A–C). However, curcumin treatment considerably prevented the increase of both UCP2 and UCP3 in post-resuscitation cardiomyocytes (Figures 3A–C). Furthermore, the level of mtTFA was significantly declined in the curcumin and control groups after reperfusion, whereas the curcumin group displayed a higher level of mtTFA compared with the control group (Figures 4A,D).

### Curcumin Attenuated Myocardial Apoptosis After Successful Resuscitation

**FIGURE 5:**
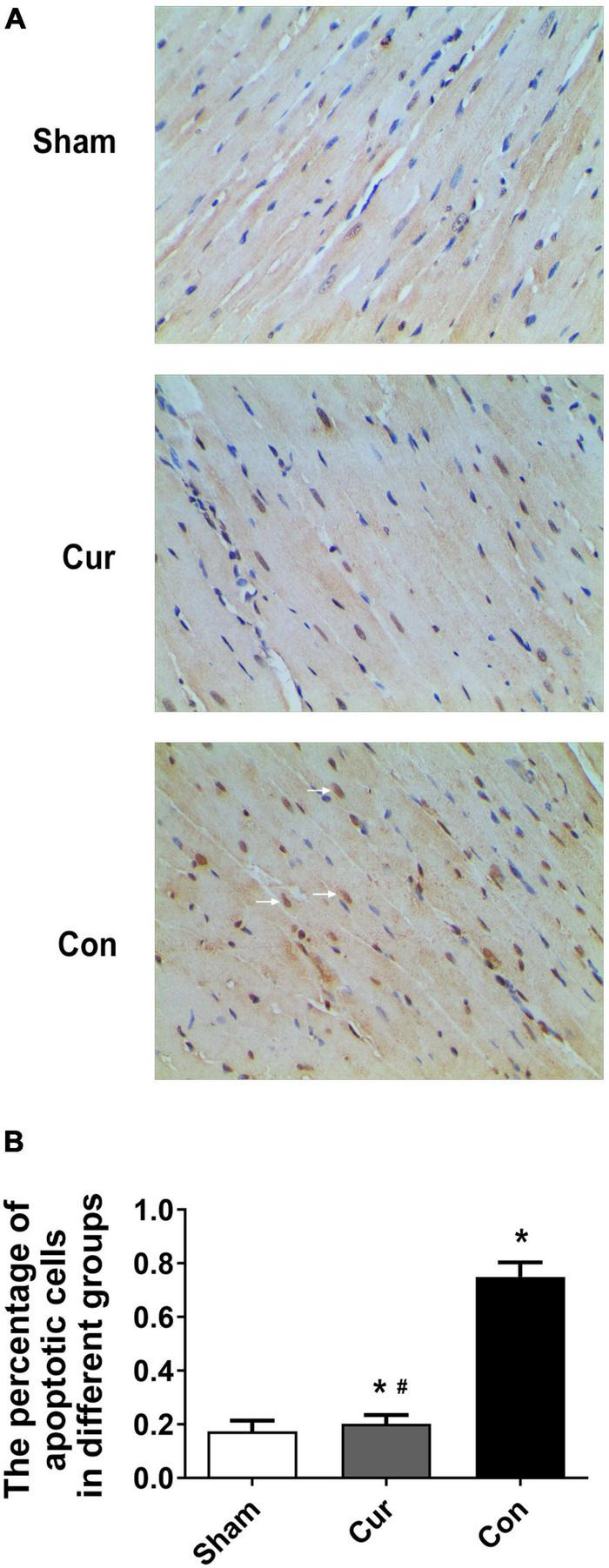
Curcumin treatment attenuated myocardial apoptosis. Myocardial apoptosis was analyzed by TUNEL staining. **(A)** Staining of the apoptotic cells. The white arrows represented the apoptotic cells. **(B)** Percentage of apoptotic cells in different groups. Cur: curcumin; Con: control. *p < 0.05 vs. the Sham ^#^*p* < 0.05 vs. the Con.

**FIGURE 6:**
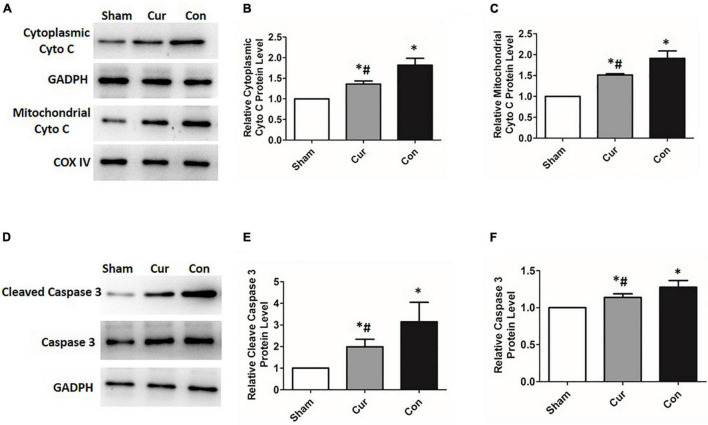
Curcumin suppressed the activation of the mitochondrial apoptosis pathway. Relevant expressions of the molecules correlated with the mitochondrial apoptosis pathway were detected by western blotting. **(A–C)** Relative expression of the protein level of Cyt C. **(D–F)** Relative expression of the protein level of caspase-3 and its cleavage. Cur: curcumin; Con: control; Cyt C: cytochrome C; *p < 0.05 vs. the Sham; ^#^*p* < 0.05 vs. the Con.

To explore the effects of curcumin on myocardial apoptosis, the percentage of apoptotic cells was detected. Compared with the sham group, cell apoptosis in the control group was distinctly increased (*P* < 0.05, Figure 5). However, curcumin treatment immensely attenuated cell apoptosis (Figure 5). We further checked the relevant molecules implicated in the mitochondrial apoptosis pathway. Cyt C in mitochondria and cytosol was measured separately. Compared with the sham group, there was a significant activation of Cyt C in both the curcumin and control groups, while its activation and release were restrained in the curcumin group in contrast to the control (Figures 6A–C). The expression of caspase-3 and its cleavage were also significantly intensified after resuscitation, and both of their expressions were depressed in the case of curcumin administration (Figures 6D–F).

## Discussion

Survival from CA predominantly depends on successful reestablishment of cardiac activity. Nevertheless, restoration of organ function after resuscitation is equally crucial on account of the concomitant activation of multiple fatal pathogenic mechanisms (6, 25). Myocardial I/R injury, an essential determinant of cardiac dysfunction and organ damage, overly affects the progression and prognosis of the patients post resuscitation (16). In this study, curcumin was applied as an auxiliary measure for the treatment of post-resuscitation myocardial dysfunction in a rat model of CA-CPR, and the plausible mechanisms were further investigated.

It was found that heart function of the rats was tremendously impaired after CA-CPR in the control group, and no rats were ultimately survived after successful resuscitation. Contrarily, curcumin administration after CPR significantly repaired myocardial dysfunction, ulteriorly prolonging the survival duration of the post-resuscitation rats. In view of this, series of indexes were detected in order to validate curcumin evoked effects and its functional mechanisms.

The potent protective effects of curcumin against I/R injury have been verified in several recent studies (26, 27). Evidences suggest that curcumin possesses the capacity of anti-oxidation, which is the key for its protective potential (11, 28). MDA, a product of oxidation, can be employed as a biomarker of I/R injury. Additionally, SOD is a vital endogenous antioxidant enzyme. Elevation of the SOD level implies the augmentation of the scavenging abilities of the oxygen free radicals (28–30). In this work, significant differences could be observed in MDA and SOD levels between the curcumin and control groups. Both the production of MDA and elimination of SOD were diminished after the curcumin treatment, which definitely supported the antioxidant property of curcumin in the rat model of CA.

As mitochondria performs a critical role in the regulation of energy metabolism and myocardial apoptosis after I/R (31–33), mitochondrial structure and function post successful resuscitation were further examined. Mitochondrial structure integrality is a prerequisite for the maintenance of mitochondrial function (34, 35). In this study, it was discovered that mitochondria were disrupted and swelling, with the dissolution of cristae in the control group. However, mitochondrial morphology almost retained normal, with well-organized cristae in the rats treated with curcumin. Besides, the respiratory function of mitochondria was partially recuperated in the curcumin group, embodied in the increased level of ATP produced by mitochondria. The opening of mPTP lead to mitochondria swelling, and the loss of ΔΨm is intimately associated with abnormal structure of mitochondria (36–38). In the present work, opening of mPTP and the collapse of ΔΨm could also be observed after resuscitation, whereas curcumin administration availably prevented these adverse effects triggered by I/R injury. Based on the aforementioned results, the latent molecular mechanisms were further probed into. Uncoupling proteins (U) are mitochondrial inner membrane proteins that are intimately associated with mitochondrial metabolism and cardioprotection (39). They can impact the proton electrochemical gradient, which is built up by the mitochondrial respiratory chain (39, 40). The role of UCP2 and UCP3 in mitochondrial defense against oxidative stress has been widely accepted, and the relevant effects is strongly correlated with the reduction of ΔΨ (41, 42). Emerging evidence has also revealed that UCP2 can weaken ΔΨm, which is subsequently interconnected with the status of mPTP (38, 39). In this condition, we inspected the expression level of UCPs, including UCP2 and UCP3. We found that UCP2 and UCP3 were significantly increased in the control group, while curcum treatment reversed these changes, indicating that UCPs might be involved in the protective functions of curcumin. Another visible ultrastructural alteration was the reduction of mitochondria numbers in the control group. mtTFA is an mitochondrial DNA (mtDNA) packaging protein that dominates mtDNA replication and transcription (43, 44). Derogation of mtTFA could bring out exhaustion of mtDNA and cause energy metabolism disorders (43–45). In addition to the protective role in mitochondrial DNA, mtTFA is also a regulator of calcium and ROS production (46). Overexpression of mtTFA can inhibit ROS production and confront oxidative stress, contributing to structural and functional stability of cardiomyocytes under the pathological circumstances (47). In the current study, the expression of mtTFA was examined afterward. It was revealed that mtTFA level was degraded in both the curcumin and control groups after resuscitation, while curcumin treatment inverted the descending trend of mtTFA. All these results sustained that curcumin participated in the mediation of mitochondrial energy metabolism and oxidative stress *via* regulating mtTFA in cardiomyocytes post-resuscitation.

The opening of mPTP and loss of ΔΨm are early signs of mitochondrial apoptosis pathway. Previous data manifest that curcumin treatment can modulate cell apoptosis through the mitochondrial pathway (48, 49). In the current work, we found that myocardial apoptosis was increased after resuscitation, and it was greatly repressed by curcumin treatment. To investigate whether the mitochondrial apoptosis pathway was implicated in curcumin mediated effects, we further measured the activity of Cyt C. Cyt C, which is located in mitochondria, can be released into the cytosol when there is a loss of ΔΨm. Aggregation of Cyt C consequentially stimulates the activation of caspases, eventually resulting in cell apoptosis (10, 50). In this study, high levels of Cyt C, caspase-3 and its active cleavage were detected in the cardiomyocytes after resuscitation, while curcumin administration could significantly curtailed the production and release of Cyt C, which finally abated the activation of caspase-3. These results corroborated the role of curcumin in regulating the mitochondrial apoptosis pathway after resuscitation in the rat model of CA.

The present work has confirmed curcumin as a candidate for the treatment of post-resuscitation myocardial dysfunction in a rat model of CA-CPR. Compared with other previous studies, this study excavated the molecular mechanism of the function of curcumin in mediating the mitochondrial metabolism and apoptosis in myocardial dysfunction after CA. Development of the target drugs based on these molecules and further conduction of relevant clinical studies will greatly increase the therapeutic tools available to attenuate myocardial I/R injury and dysfunction of cardiomyocytes after CA.

## Conclusion

Curcumin ameliorates myocardial dysfunction and improves the outcome of CPR in the rat model of CA. It could function through anti-oxidation, protection of the mitochondrial structure and energy metabolism, as well as the suppression of the mitochondrial apoptosis pathway. Molecules correlated with mitochondrial metabolism and apoptosis, including UCPs, mtTFA, Cyt C and caspase-3, were involved in the curcumin mediated protective effects. Further investigations of these molecules might afford new strategies for the treatment of myocardial dysfunction after CA.

## Data Availability Statement

The original contributions presented in the study are included in the article/supplementary material, further inquiries can be directed to the corresponding authors.

## Ethics Statement

This study adhered to ethical standards for animal research and was approved by the Animal Care and Use Committee of the Sun Yat-sen University.

## Author Contributions

JH and ZY: study design. JZ and LJ: animal model construction. SL: physiology and biochemistry measurements and statistical analysis. JH and JZ: detection of apoptosis and molecular biological indexes and writing of the manuscript. All authors read and approved the final manuscript.

## Conflict of Interest

The authors declare that the research was conducted in the absence of any commercial or financial relationships that could be construed as a potential conflict of interest.

## Publisher’s Note

All claims expressed in this article are solely those of the authors and do not necessarily represent those of their affiliated organizations, or those of the publisher, the editors and the reviewers. Any product that may be evaluated in this article, or claim that may be made by its manufacturer, is not guaranteed or endorsed by the publisher.
